# Efficacy and Safety of Fractional Q‐Switched Ruby Laser for Nevus of Ota in an Infant— Case Report

**DOI:** 10.1111/jocd.70386

**Published:** 2025-08-12

**Authors:** Rhea Ahuja, Patrick Po‐Han Huang

**Affiliations:** ^1^ Visiting Fellow in Aesthetic Dermatology Huang PH Dermatology and Aesthetics Kaohsiung Taiwan; ^2^ Dermatologist Huang PH Dermatology and Aesthetics Kaohsiung Taiwan

**Keywords:** early intervention, fractional Q‐switched ruby, infant, nevus of Ota, safety

To the Editor,

Nevus of Ota is a dermal melanocytic hamartoma characterized by bluish‐gray pigmentation along the V1 and V2 distributions of the trigeminal nerve. Present at birth in about 60% of cases, it is more common in Asian and dark‐skinned individuals [1]. Treatment is often delayed until adolescence [2], by which time the pigmentation has already left a lasting impact. Not only does early intervention improve treatment efficacy, but it also prevents the long‐term psychosocial burden associated with visible facial pigmentation. Here, we report the remarkable efficacy and minimal downtime of fractional Q‐switched ruby laser in an infant, underscoring the benefits of timely intervention.

A 6‐month‐old female infant with Fitzpatrick skin type III presented with bluish‐black hyperpigmentation affecting the right ophthalmic and maxillary segments of the face. To determine the optimal laser approach, we conducted a comparative evaluation on distinct, adjacent areas of the nevus of Ota located on the right forehead of the infant. The treated zones received either Q‐switched ruby laser (694 nm, Q Plus R, Quanta System S.p.A., Samarate, Italy) in flat‐optic mode (QR) at 1.3 to 2.8 J/cm^2^, fractional mode (FQR) at 0.62 J/cm^2^, or picosecond Nd:YAG laser (1064 nm, Discovery Pico, Quanta System S.p.A., Samarate, Italy) in fractional mode (FPN) at 0.20 J/cm^2^ (Figure 1a). Upon follow‐up after a week, the QR‐treated area developed a crusted lesion, whereas the FQR‐ and FPN‐treated areas showed some improvement without significant epidermal damage or crusting (Figure 1b).

**FIGURE 1:**
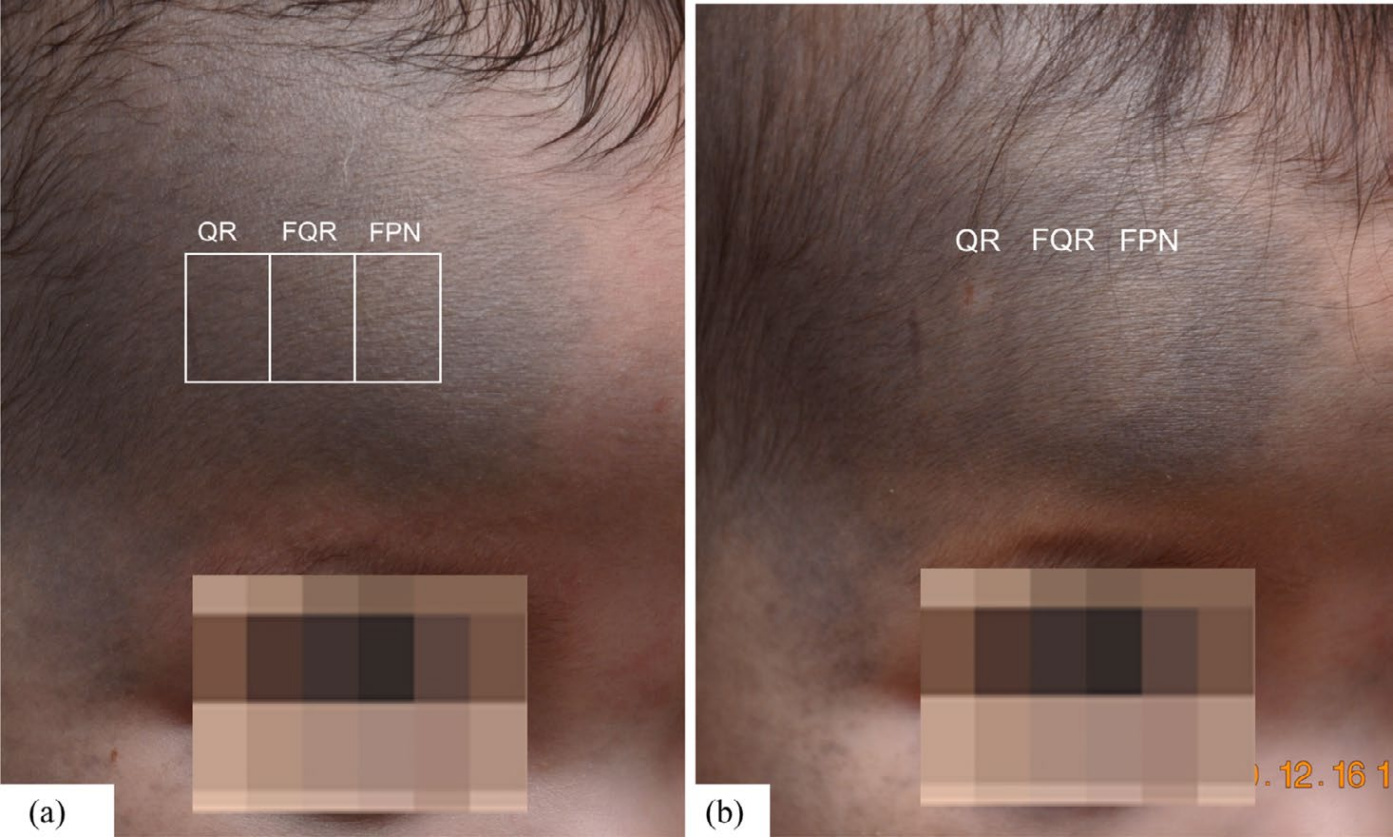
(a) To determine the optimal laser treatment approach, we compared a Q‐switched ruby laser (QR), a fractional Q‐switched ruby laser (FQR), and a fractional picosecond Nd:YAG laser (FPN) to treat a designated area on the right side of the forehead. (b) At 1‐week follow‐up, a crusted spot was observed in the QR‐treated area, whereas the FQR‐ and FPN‐treated areas showed improvement without significant epidermal damage.

We further proceeded with increased fluence for both FQR at 0.94 J/cm^2^ and FPN at 0.35–0.45 J/cm^2^ and conducted an additional comparative treatment on the right cheek (Figure 2a). Immediately post‐procedure, pinpoint bleeding was observed in the FPN‐treated area, possibly due to its shorter pulse duration and higher peak power, leading to greater photomechanical effects and vascular disruption (Figure 2b). After 2 weeks, the FQR‐treated area showed more pronounced improvement compared to FPN (Figure 2c). Consequently, after preliminary testing, we proceeded with FQR (0.94 J/cm^2^, fractional 9 mm High Coverage Handpiece) with non‐overlapped sweeping, for the entire affected area. Each session was performed under topical anesthesia using a thin layer of EMLA cream, ensuring a largely pain‐free experience. Only mild and temporary post‐procedural erythema was observed. A total of six treatment sessions were conducted at 6‐week intervals, resulting in a remarkable 75%–90% improvement on photographic comparison (Figure 3).

**FIGURE 2:**
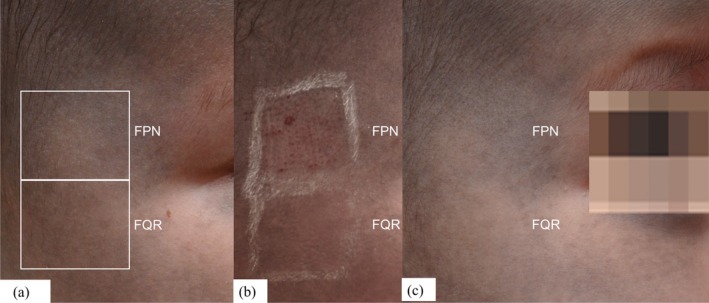
(a) An additional comparative treatment conducted on the right cheek with increased fluences for both FQR and FPN. (b) Immediately post‐procedure, pinpoint bleeding was observed in the FPN‐treated area (c) After 2 weeks, the FQR‐treated area showed more pronounced improvement compared to FPN.

**FIGURE 3:**
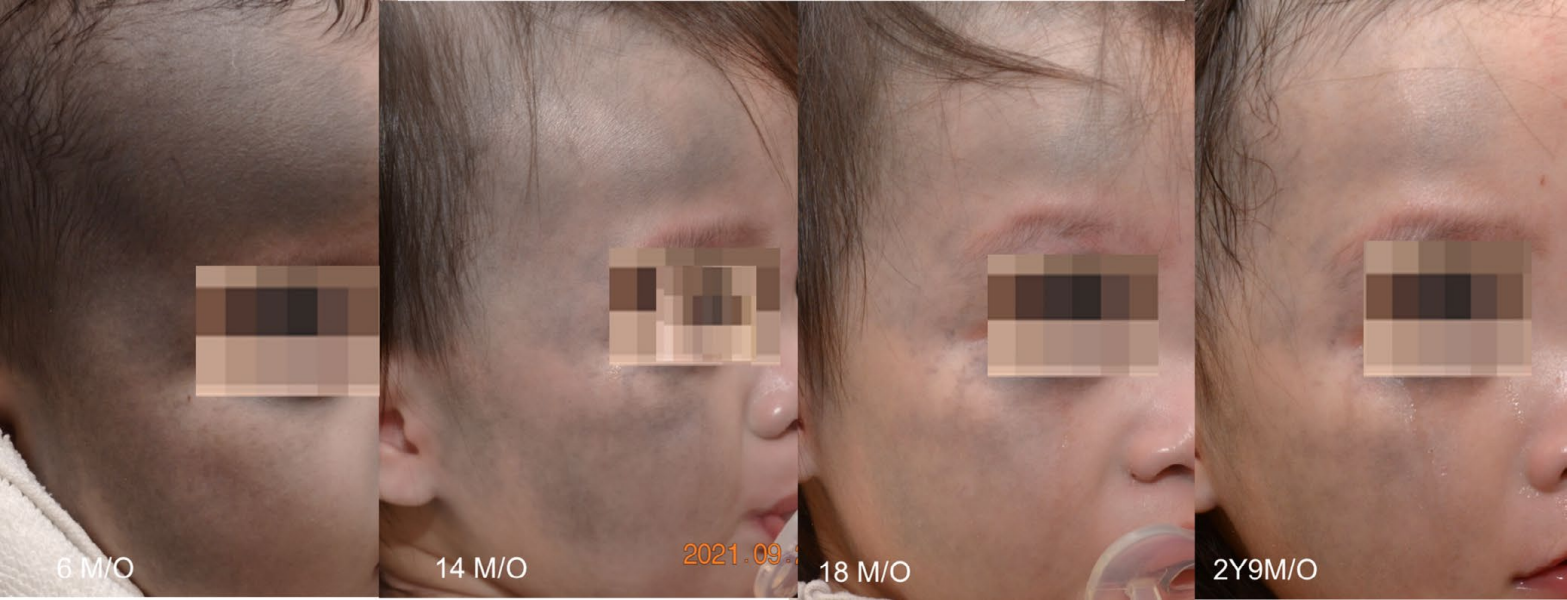
Remarkable improvement with significant fading of color without residual hypopigmentation after six sessions with FQR.

Melanin absorption is highest in the visible light spectrum, peaking in the red wavelength range of ruby lasers, with a sharp decline beyond 800 nm. This makes the ruby laser more effectively and selectively absorbed by melanin than Nd:YAG, allowing for greater response even at lower fluences [1] [3]. In lighter skin types, such as our patient's, the risk of hypopigmentation due to competing epidermal chromophores is reduced. This risk was further minimized by using a fractional ruby laser, which delivers less than 40% skin coverage per pulse [4], reducing epidermal injury while ensuring effective pigment clearance. The fractional mode also creates microscopic treatment zones, preserving untreated areas that facilitate faster healing and shorter recovery times. Previously, FQR has been used for acquired bilateral nevus of Ota‐like macules (ABNOM) [5], but its response in ABNOM has been less favorable with a greater number of sessions and risk of postinflammatory hyperpigmentation (PIH). The differences in response may be attributed to distinct melanocyte origins and distribution patterns—nevus of Ota melanocytes are of neuroectodermal origin and evenly distributed in the dermis, whereas ABNOM melanocytes are more perivascular and influenced by external stimuli like hormones and trauma [6].

For pain management, a thin layer of EMLA was used after informed parental consent. While not formally approved for infants, extensive studies have demonstrated its safety, with doses up to 2 g for 4 h causing no clinically significant increase in methemoglobin levels in infants aged 3–12 months, supporting its use for procedural pain relief [7]. Importantly, laser sessions are more easily performed in infants, who remain still with topical anesthesia, compared to older children, where procedural distress often necessitates general anesthesia [8].

The psychological impact of visible pigmentary disorders in childhood is often underestimated. Chronic skin conditions, including birthmarks, contribute to social stigma, affecting self‐perception and emotional well‐being [9]. Early Q‐switched ruby laser treatment for nevus of Ota has demonstrated higher clearance rates in preschool children (93.1%) with fewer sessions (3.6 vs. 5.1), fewer adverse reactions (4.7% vs. 13.7%), and better psychological outcomes (posttreatment CDI score 10.8 vs. 13.6 in older children) [10]. These findings reinforce the need for timely intervention to optimize both clinical and psychosocial outcomes.

Thus, treatment with FQR laser was not only well tolerated by our patient but also led to significant improvement with no major posttreatment adverse effects and high parental satisfaction. This case highlights the importance of early intervention not only in enhancing aesthetic and psychological outcomes but also in shaping future protocols for pediatric pigmentary disorders. It underscores the potential role of fractional Q‐switched ruby laser as a safe and effective option in infancy, warranting future research.

As this is a single‐case report, the generalizability of our findings is inherently limited. While the outcomes observed were encouraging, they should be interpreted with caution. Individual variations in lesion depth, skin type, and treatment response may influence results. Larger, controlled studies with long‐term follow‐up are needed to validate the safety, efficacy, and psychosocial benefits of early laser intervention for nevus of Ota in infants.

## Author Contributions

**Dr. Rhea Ahuja** conceptualized and designed the study, drafted the initial manuscript, and revised the manuscript. **Dr. Patrick Po‐Han Huang** conceptualized and designed the study, collected data, drafted the initial manuscript, and critically reviewed and revised the manuscript.

## Disclosure

Role of Funder/Sponsor : None.

Clinical Trial Registration: Not applicable.

## Ethics Statement

The parents of the patient have given consent for publication of the child's case details and photographs in the manuscript.

## Conflicts of Interest

The authors declare no conflicts of interest.

## Data Availability

Data sharing is not applicable to this article as no new data were created or analyzed in this study.
